# MEMS Sensor Technologies for Human Centred Applications in Healthcare, Physical Activities, Safety and Environmental Sensing: A Review on Research Activities in Italy

**DOI:** 10.3390/s150306441

**Published:** 2015-03-17

**Authors:** Gastone Ciuti, Leonardo Ricotti, Arianna Menciassi, Paolo Dario

**Affiliations:** The BioRobotics Institute, Scuola Superiore Sant’Anna, Pisa 56025, Italy; E-Mails: l.ricotti@sssup.it (L.R.); a.menciassi@sssup.it (A.M.); p.dario@sssup.it (P.D.)

**Keywords:** MEMS sensor technologies, human centred applications, research activity in Italy, healthcare, rehabilitation, physical activities, sport science, safety, environmental sensing

## Abstract

Over the past few decades the increased level of public awareness concerning healthcare, physical activities, safety and environmental sensing has created an emerging need for smart sensor technologies and monitoring devices able to sense, classify, and provide feedbacks to users’ health status and physical activities, as well as to evaluate environmental and safety conditions in a pervasive, accurate and reliable fashion. Monitoring and precisely quantifying users’ physical activity with inertial measurement unit-based devices, for instance, has also proven to be important in health management of patients affected by chronic diseases, e.g., Parkinson’s disease, many of which are becoming highly prevalent in Italy and in the Western world. This review paper will focus on MEMS sensor technologies developed in Italy in the last three years describing research achievements for healthcare and physical activity, safety and environmental sensing, in addition to smart systems integration. Innovative and smart integrated solutions for sensing devices, pursued and implemented in Italian research centres, will be highlighted, together with specific applications of such technologies. Finally, the paper will depict the future perspective of sensor technologies and corresponding exploitation opportunities, again with a specific focus on Italy.

## 1. Introduction

Micro-Electro-Mechanical Systems (MEMS) are mechanical and electro-mechanical elements (*i.e.*, devices and structures) developed through microfabrication techniques. MEMS, also referred to microsystems technology (MST) in Europe or micromachined devices in Japan, is probably not a fully appropriate term, as not all the current so-called MEMS, MST or micromachined devices are “electromechanical” modules and/or “systems”. Nevertheless, the definition is now widely applied to several miniaturised devices, *i.e.*, generally three-dimensional microstructures mostly made of silicon and obtained by isotropic and anisotropic etching, various thin film deposition methods, anodic bonding and masking and doping techniques normally employed in integrated circuit (IC) manufacture [1,2].

The origins of what we call nowadays “MEMS technologies” can be traced back to 1954, when a paper by Smith entitled “Piezoresistive effects in silicon and germanium” was published in the international journal “*Physical Review*”. It described, for the first time, stress sensitive effects in silicon and germanium, termed “piezoresistance” [3]. Smith’s paper was followed in 1955 by probably the first publication that assessed the possibility to replace bulky electromechanical sensors with small devices [4]. Throughout the early 1960s, different manuscripts from the Honeywell Research Centre and the Bell Labs reported and described the first silicon diaphragm pressure sensors and strain gauges [5,6]. In these years, interest in silicon sensor technologies grew dramatically, and by the late 1960s a number of US companies, pioneers and leaders in this field, commercialised the first silicon pressure sensors. In the early 1970s, developments in micromachining and improvements of silicon processing brought to pressure sensors with non-planar diaphragm geometries, showing superior performance; these were arguably the first proper MEMS sensors [7]. Since then, MEMS technologies have progressively established a wide range of small, high performance and often inexpensive sensors able to sense and thus respond to many physical variables (e.g., pressure, position, motion, strain, radiation and flow), from low volume/high cost products in industry and aerospace (1970s to 1980s) to ultra-high volume/very low cost products in consumer electronics (early 2000s onwards) [8,9].

Nowadays, MEMS-based devices range from simple arrangements with no moving parts, to complex electromechanical systems with several moving elements under the control of integrated microelectronics. It is worth mentioning that MEMS technology has consistently been successful in the physical sensing context (*i.e.*, exploiting the control capabilities of microsensors). Moreover, the technology advancements of MEMS sensors have been strongly pushed and move together with information and communications technologies, with the integration of low power circuits, wireless communication modules and wireless sensor networks, enabling the design of compact, high performance, low power and low cost solutions for a wide range of applications [10,11].

In a global MEMS market, expected to reach one trillion units per year within the next decade, several applications and scenarios are nowadays leading the scene in industry and research. This paves the way to the continuous integration and development of smart MEMS technologies able to merge the measuring capabilities with other key features, such as digital signal processing and elaboration for embedded intelligence.

In this context, the healthcare and well-being domain represents one of the most attractive sectors with a high potential to contribute to the market growth and development of new MEMS sensor technology. Portable, disposable and wearable sensors for healthcare, but also for sensing physical activities and for well-being in general are used for monitoring, e.g., heart rate, blood pressure, breath, and to perform specific disease diagnoses; they also include systems to care for a growing aging population and chronically ill patients. A significant indicator of the massive trend in MEMS sensor technologies in the healthcare and well-being domain is represented by the continuous emergence of novel medical devices. For instance, the market of disposable medical devices embedding MEMS sensors for monitoring and diagnosis is forecast to rise more than 6 billion dollars in 2018. The use of sensors to monitor chronic diseases, such as hypertension, obesity, diabetes, sleep disorders and heart failures, is the key-element to maintain high the quality of life (often predicting the event) and also to reduce the cost of healthcare thanks to a remote monitoring; moreover, early intervention is vital for patients at risk of developing chronic diseases [12].

New and emerging applications are also associated to effective environmental sensors. Monitoring of structural integrity of vehicles, such as for composites used in aircraft, and buildings is just one example of the huge exploitation and diffusion of MEMS sensor technologies in this sector. Another major application will be in so-called “smart cities”, involving, e.g., active traffic management and interactive transportation systems, smart grids for lighting and electricity supply, high spatial/temporal resolution pollution monitoring and weather forecasting, most of them expected to be enabled by wireless sensor networks and clouds. Finally, other interesting scenarios where MEMS sensors are widely employed are in communication processes in general, supply of utilities, food industry, farming, media and gaming [8,13].

In recent years, Biomedical or Biological Micro-Electro-Mechanical Systems (BioMEMS) have shown a tremendous potential for the biomedical field, both from a research and industrial point of view. The most promising application domains concern, advanced diagnosis, therapy, and tissue engineering strategies. In the area of biomolecular analysis and sensing, BioMEMS currently play a significant role, providing platforms for sensing microorganisms, DNA strands, molecules, viruses and cells [14,15].

In this emerging framework, this review paper is aimed at presenting MEMS sensor technologies developed by Italian research centres in the last three years, describing research implementations and developments for specific emerging application fields, such as: (i) healthcare (Section 2.1) and (ii) physical activities, safety and environment sensing (Section 3). Finally, perspectives of MEMS sensor technologies and their possible exploitation with a focus on Italy will be discussed in Section 3.

## 2. MEMS-Based Sensor Technologies for Human Centred Applications

### 2.1. Healthcare

Sensor technologies in the healthcare domain range from physiological monitoring, such as heart rate, to screening applications, such as blood analysis, to falls risk applications, assistance and rehabilitation. Both at home and in outdoor environments, telehealth, telemonitoring, and mobile health (mHealth) sensor technologies enable remote monitoring and management of patients affected by chronic diseases, including: (i) diabetes; (ii) congestive heart failure; and (iii) obstructive pulmonary disease.

Key factors for the proliferation of sensors in medical healthcare are the availability of low cost microsystem sensor technologies (e.g., MEMS) coupled, in many cases, with low cost, low power microcontrollers and efficient and reliable telemetry modules. These aspects have enabled the development of compact, reliable, robust, accurate and low power solutions.

Sensors used in hospital and healthcare facilities focus on medical screening and diagnostic applications, such as the point-of-care parameters measurement device. Novel sensors for human biomedical signal acquisition, together with wireless connectivity and low power solutions, are generating new opportunities for wearable devices, which enable and guarantee continuous monitoring together with users’ freedom of movement. There is also a growing interest on diagnostic MEMS sensors that perform cholesterol monitoring, pregnancy monitoring, food allergy detection, and DNA analyses. In many cases, sensor technologies can deliver significant results, representing a key element to assist decision-making prior to seeking formal clinical intervention and care. In this framework, the design and development of technologies for healthcare has attracted the attention and interest of several research centres.

#### 2.1.1. Medicine

MEMS-based sensors have recently emerged as a key element in medicine. In this context, inertial measurement units (IMU (inertial measurement unit), composed by accelerometers and gyroscopes) dominate the stage, with several application domains.

**Figure 1:**
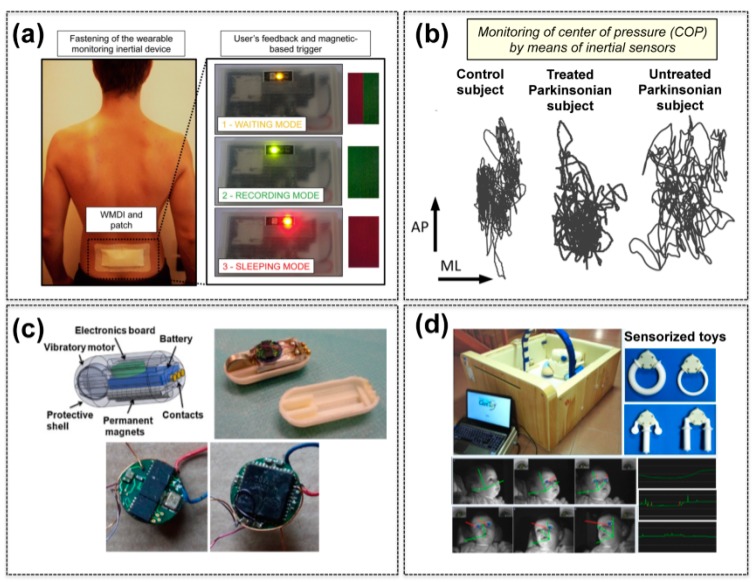
Examples of MEMS for medical applications. (**a**) Wearable monitoring inertial device for measuring sexual performance. Adapted with permission from [16] (copyright: Elsevier); (**b**) Center of pressure displacement maps obtained by means of tri-axial accelerometers mounted on healthy and Parkinsonian subjects. AP = antero-posterior plane, ML = medio-lateral plane. Adapted with permission from [24] (copyright: Creative Commons); (**c**) Endoscopic capsules provided with inertial sensors for vibratory motor control. Adapted with permission from [34] (copyright: Elsevier); (**d**) MEMS integrated in toys for monitoring preterm infants at risk of neurodevelopmental disorders. Adapted with permission from [38] (copyright: Creative Commons).

Fundamental studies have been conducted in sexual medicine. A low invasive wearable platform equipped with a tri-axial MEMS accelerometer has been developed by Ciuti *et al.* [16] for monitoring movement parameters during sexual intercourse. The system, named HuMOVE, enables quantitative measurement of inertial parameters during the sexual activity, meeting requirements of data storage, sampling rate, wearability, and interfacing methods, which are critical for human sexual intercourse performance analysis (Figure 1a). A Hall-effect magnetic sensor is embedded in the HuMOVE platform and it is exploited for switching the device on and off through a magnetic-based credit card-shaped device. Preliminary experimental tests, performed during simulated intercourse conditions on human model, confirmed the accuracy of the sexual performance evaluation platform and the effectiveness of the movement derived parameters. The HuMOVE system demonstrated to have the potential to be a helpful tool for physicians to accurately classify sexual disorders (e.g., premature or delayed ejaculation).

A significant number of studies have been already performed on Parkinson’s disease (PD) assessment, considered a model disorder for motor impairment [17]. In general, motion sensors, such as accelerometers and gyroscopes, are used in combination with light, usually flexible, and comfortable electronics that do not interfere with normal human motion and activities. A fundamental advantage in comparison with traditional clinical assessment systems is that these sensors guarantee a more objective, quantitative, and reliable assessment of symptoms; they also show significant advantages compared to in-lab technologies (e.g., optoelectronic motion capture) as they allow long term monitoring in real life scenarios. In particular, quantitative assessment strategies allow users, clinicians, and scientists to gain more objective, unobtrusive, and relevant data out of their clinical evaluation for a pervasive (*i.e.*, everywhere) and intensive (*i.e.*, anytime) tools for ambulatory assessment and even rehabilitation of motor and (partly) non-motor symptoms in PD [18].

Tremors, typical of PD, have been studied by Di Pino *et al.* [19]. In this study, the authors introduced the neurophysiological substrate of tremor and presented a proof-of-concept of tremor assessment for diagnostic support and drug efficacy in Parkinsonians and, in general, in subjects affected by essential tremor. Results were achieved through a self-assembled wireless, low cost wearable device, with an embedded tri-axial MEMS accelerometer and designed for operating in patient natural environment.

Mellone *et al.* used a Hilbert-Huang transformation (HHT) on a set of postural parameters extracted from acceleration signals for tremor removal in patients with disorders of the central and peripheral nervous system, such as PD. HHT, with respect to a linear low pass filter, demonstrated the advantage of providing a filtering tool, without *a priori* knowledge, which efficiently managed the nonlinear and non-stationary interference due to tremor; beyond tremors, it gives descriptive measures of postural function [20].

Studies on postural control in Parkinsonian subjects with the use of inertial sensors have been performed by Mancini *et al.* [21,22,23] and Maetzler *et al.* [24] (Figure 1b). An instrumented Timed Up and Go (iTUG) test was used by Palmerini *et al.* to acquire quantitative information about the TUG performance of PD subjects; the TUG test is a widely accepted clinical test used to assess mobility in PD. In particular, a single tri-axial MEMS accelerometer, placed at the lower back, was used to record the acceleration signals during the test with the aim to select reliable measures to recognise and quantify differences between the motor patterns of healthy and PD subjects. A subset of three features (*i.e.*, two from turning, and one from the sit-to-walk component), combined with an easily interpretable classifier (*i.e.*, linear discriminant analysis), resulted to have the finest accuracy in discriminating between healthy and early-mild PD subjects [25,26].

Accelerometric parameters of gait under different neurological conditions compared to the gait of healthy subjects have been studied by Fazio *et al.* [27]. Seventeen patients affected by PD, 24 subjects with ataxic gait due to different diseases, and 24 healthy subjects were analysed with a tri-axial MEMS accelerometer (with a portable datalogger), which measured acceleration and deceleration on an anterior-posterior, mediolateral and vertical plane at an approximate level of the centre of mass (*i.e.*, back sacral localization), and in other two positions (*i.e.*, sternal and frontal sacral region) during a steady-state walking. A significant reduction of acceleration parameters in neurological patients compared with healthy subjects was observed, suggesting that a tri-axial accelerometer system can represent a practical and low cost tool for assessing the alteration of perambulation.

Finally, an inertial-based system for motion analysis, based on 9-axis complete MEMS inertial modules fixed on the fingers and forearm, has been developed by Cavallo *et al.* for evaluating motor skill performance and deficits in hands due to strokes or diseases of various clinical natures. The technological solution, tested in the study of PD, is able to track the users’ hand motions in real time and send data through wireless communication, with the benefit of reducing the clutter and the disadvantages of a glove equipped with sensors [28].

Long term recording of biomedical signals, such as electrocardiogram (ECG), electromyogram (EMG), respiration and other information (e.g., body motion) can improve diagnosis and potentially monitor the evolution of many prevalent diseases. However, as previously highlighted, long term monitoring requires specific solutions (*i.e.*, portable and wearable equipment) that should be particularly comfortable for patients. The key issues of portable biomedical devices are: (i) power consumption; (ii) long term sensor stability and effectiveness; (iii) comfortable wearing; and (iv) wireless connectivity.

An accelerometer-based device suitable for long term monitoring of the breathing and heart rates (HR), along with postural changes during sleep and wakefulness, was presented by Lapi *et al.* [29]. Recordings of respiratory frequency, HR, posture and voluntary cough were obtained from a group of volunteers who also participated in sleep studies. A pair of tri-axial capacitive MEMS accelerometers was positioned at the level of the 10th rib along the mid-axillary line bilaterally for simultaneous recordings of respiratory movements, HR and body position. Tests performed in comparison, when possible, with conventional spirometry demonstrated the accuracy of the system for the monitoring of respiratory movements as an important feature in medical care planning.

In the long term monitoring scenario, biopotentials and body movements can be recorded using textile electrodes embedded in clothes. Bifulco *et al.* developed a sensorized garment equipped with low power electronics for signal acquisition and data wireless transmission via the Bluetooth communication protocol. A small, battery powered, biopotential amplifier and tri-axial acceleration body monitor was realized, which was comfortable to wear for patients [30]. A similar approach for a multi-parameter wireless shirt for physiological monitoring was presented by Sardini *et al.* [31], also in the field of health status monitoring of elderly patients or patients undergoing home therapy. The research activity concerns the development of a new wearable device that can monitor several physiological parameters of a person in a non-invasive manner, such as (i) ECG; (ii) HR; (iii) respiratory rate and (iv) tri-axial motion (*i.e.*, acceleration and position) of the subject measured using a MEMS accelerometer.

MEMS accelerometers have been also applied to capsule endoscopes for localization purposes during diagnostic procedures [32,33]. Moreover, Ciuti *et al.* [34] presented a capsule prototype integrating a permanent magnet with a vibrating motor and a tri-axial accelerometer, along with an electronic module that allows remote control of the motor and wireless transmission of the inertial data to a host PC (Figure 1c). *Ex-vivo* tests were performed assessing the efficacy of vibrations in friction reduction, as well as the appropriateness of the inertial sensing scheme in capturing the characteristics of the capsule vibrations; these findings may be exploited for the on-the-fly adjustment of the vibratory motor frequency, based on accelerometer data, in order to adaptively minimize the capsule friction during a capsule-based endoscopic procedure.

Novel approaches for measuring infants’ manual actions have been supported by sensorized MEMS-based platforms usable in natural settings, such as sensorized wireless toys that can be exploited for diagnosis and, in some cases, rehabilitation purposes. The study and measurement of manual actions and forces in infants can provide understandings on the typical and atypical motor development. A sensorized wireless toy has been developed with embedded MEMS pressure sensors and audio/visual feedback, *etc.* [35]. In the study of Serio *et al.*, infants showed a good grade of acceptance to such kind of sensorized toys, as confirmed by the results of preliminary tests that involved nine healthy infants; in particular: (i) the dimensions matched infants’ anthropometrics; (ii) the device was robust and safe; (iii) the acquired signals were in the expected range and (iv) the wireless communication was stable [36]. Further improvements of these technologies have been performed, within the EU CareToy Project [37], with a new modular MEMS-based system for intensive, patient specific, home-based and family centred early intervention, managed remotely by rehabilitation staff. A randomized controlled trial has been designed to evaluate the efficacy of training in a first sample of low risk preterm infants, as an innovative and effective tool for early intervention in preterm infants [38] (Figure 1d).

In cardiovascular medical procedures and specifically in the framework of the EU SensorART Project [39], a sensorized ventricular assist device has been developed in order to improve both the value and quality of patients’ treatment and the operating workflow of medical specialists. The SensorART platform comprises both implantable and wearable devices that are exploited to assist and support control algorithms by gathering physiological/physics parameters using a non-invasive approach. In this scenario, a MEMS pressure sensor, produced by STMicroelectronics (Geneva, Switzerland [40]), has been integrated in a wearable and implantable platform (blood pressure catheter) for monitoring the arterial blood pressure, together with a 3D accelerometer for deriving the posture and the activity of the patient [41]. In further developments of the SensorART platform, the aforementioned pressure sensor-based system for the implantable catheter has been replaced with two catheters with piezoresistive tips (Mikro-Tip^®^ by the Millar Instruments, Houston, TX, USA), positioned at the pump input and output, respectively [42].

MEMS technology has found important applications in the medical ultrasound imaging ultrasound imaging field. High performing and thermally efficient ultrasound probes are required to successfully enable advanced techniques; in this context, standard ultrasound transducer technology is currently the limiting factor. Capacitive micro-machined ultrasonic transducers (CMUTs) are MEMS-based devices that have been widely recognized as a valuable alternative to piezoelectric transducer technology in a variety of medical imaging applications. Advantageous factors that may justify the adoption of this MEMS technology in the medical ultrasound imaging field are: (i) wideband operation; (ii) good thermal efficiency and (iii) low fabrication cost, especially for those applications requiring a high volume production of small area dies. A significant study has been conducted by Savoia *et al.* [43] that reported the design, development, and characterization of a 12-MHz ultrasound probe for medical imaging, based on a CMUT array.

Lorentz-force-based MEMS magnetometers represent a promising solution for the development of sensor technologies for several medical applications. Lorentz force devices can achieve better performance in terms of minimum detectable magnetic flux density per unit current consumption and bandwidth [44]; they can provide the prospect of driving more magnetometers in series through a single current source, thus enabling the fabrication of tri-axis low power magnetic field sensors [45].

A low cost integrated microsensor composed by an antenna-coupled microbolometer as detector has been developed and presented by Perenzoni *et al.* for biomedical applications. For this specific technology, the detector and the IC have been fabricated in a custom MEMS technology and a standard complementary metal-oxide semiconductor (CMOS) technology, respectively, thus demonstrating proper operating functionalities in first electrical measurements [46].

In recent years, the biological and biomedical employment of micro- and nanotechnology, commonly referred to BioMEMS, has become progressively prevalent and has found a widespread use in a variety of applications, such as diagnostics, therapeutics, and tissue engineering, both from a research and industrial point of view. Tedeschi *et al.* proposed a resonant mass sensor based on a CMOS-compatible MEMS technology, targeted at the label-free selective detection of biomolecules (*i.e.*, specifically RNA sequences) [47]. A novel, completely transparent BioMEMS device has been devised and manufactured using finite element analysis and micro-fabrication techniques by Fior *et al.* [48]. The device, based on a silicon dioxide-silicon nitride structure, has been designed to be employed and used for testing the mechanical properties of single living cells and it demonstrated to be versatile and suitable for coupling with other analysis techniques.

#### 2.1.2. Assistance and Rehabilitation

The ageing process of the population is increasing interest in solutions to improve the quality of life of elderly or disabled people and their families, also in view of an economically sustainable healthcare system. At the global level, the proportion of 60-plus old people has risen from only 8% of the world population (200 million people) in 1950 to around 11% (760 million people) in 2011, with an even more dramatic increase still ahead as those 60-plus old people are expected to reach 22% (2 billion people) by 2050 [49]; hence, the urgency to provide solutions enabling our ageing society to remain active, creative, productive, and—above all—independent. In Italy by 2030 over 25% of the population and by 2050 over 30% is expected to be aged 65 and over [50]. In this framework, ambient assisted living, ambient intelligence, human centred assistance and rehabilitation are moving towards the development of modular, adaptable and intelligent systems and tools to cope with changing needs that characterize the life of elderlies and people with chronic or acute diseases [51]. Sensor networks and monitoring devices are enabling technologies for ambient assisted living solutions in both domestic environments and outdoor scenarios and to provide assistance and rehabilitation services.

Activity recognition systems have been demonstrated in several studies to be very effective for tracking users’ activities in healthcare, but also in assistance and rehabilitation. Several research groups have developed activity recognition systems and multi-sensor systems for detecting and classifying human activities. Mannini *at al*. developed a wearable sensor system that collects data from a single thigh-mounted tri-axial MEMS accelerometer; the system performs activity classification (*i.e.*, sit, stand, cycle, walk, run), and speed estimation for walk (*i.e.*, run) labelled data features. These tasks of classification/estimation are achieved by cascading two support vector machine classifiers. Activity classification accuracy higher than 99% and root mean square errors of 0.28 km/h for speed estimation have been obtained in preliminary experiments [52]. In this framework, significant results have been obtained by the same group, which used Markov models to discern between different human activities through accelerometers [53]; a recent study showed that, with a single accelerometer mounted on the wrist or the ankle and a specific algorithms using a limited number of features, it is possible to classify activities in four classes, with a good computational efficiency [54].

Comotti *et al.* developed a wireless and low power attitude and heading reference systems network based on low cost MEMS sensors for motion tracking systems (*i.e.*, iNEMO^TM^ M1 9-axis motion sensing system composed by a 6-axis digital e-compass, a 3-axis digital gyroscope and an ARM^®^Cortex™-M3 32-bit microcontroller—STMicroelectronics, Geneva, Switzerland) [55]. Palumbo *et al.* presented an activity recognition system that classifies a set of common daily activities carried out by the user exploiting both the data sampled by MEMS accelerometers, and the reciprocal received signal strength values coming from worn wireless sensor devices, and from sensors installed in the environment. The accelerometer and received signal strength stream were modelled using recurrent neural networks implemented as efficient echo state networks, within the reservoir computing paradigm [56]. A novel personalized recognition model framework, for physical activities, based on a semi-supervised clustering approach to avoid fixed threshold techniques and traditional clustering methods by using a single accelerometer, has been developed by Ali *et al.* It required a limited amount of data to compute the initial centroids for clustering of physical activities and achieved an accuracy of about 93% on average with the potential capability of recognizing subjects’ behavioural shifts, falls and exceptional events [57]. A smart shirt, embedding an inertial system (*i.e.*, a MEMS accelerometer), able to monitor biomedical parameters and managing some alarms for a robot walker has been presented by Dionisi *et al.* [58]. Some typical human movements have been tested and the obtained results allowed one to recognize movements and positions of a patient using the antero-posterior and medio-lateral angles derived by the acceleration signals.

Falls are one of the main causes of trauma, disability and death among old people. As demonstrated by several studies, inertial-based sensor technologies and accelerometer-based devices represent reliable tools to detect falls in controlled environments and also in outdoor scenarios, together with the implementation of specific fall detection algorithms [59].

An algorithm for feature extraction characterized by a low computational cost and the implementation of a machine learning scheme for detection of fall events in the elderly, by using a tri-axial MEMS wearable wireless accelerometer, have been presented by Rescio *et al.* [60]. The proposed approach allows generalizing the detection of fall events in several practical conditions, after a limited period for calibration. The system appeared invariant to several conditions: (i) age; (ii) weight; (iii) height of people and (iv) relative positioning area, overcoming the drawbacks of already well-established threshold-based approaches in which several parameters need to be manually estimated according to the specific features of the end user. The supervised clustering step has been achieved in the study of Rescio *et al.* by implementing a one class support vector machine classifier in a stand-alone PC and a polynomial kernel function has been used in order to limit the computational cost, while maintaining high performances in terms of reliability and efficiency [61].

A promising system for detecting and classifying human activities based on a multi-sensor approach, in which classification is mostly aimed at detecting falls, has been presented by Ugolotti *et al.* [62]. The algorithms, as well as their structure, are aimed at analysing and classifying complex movements (e.g., walking, sitting, jumping, running, falling) of multiple people at the same time. The system exploits four calibrated cameras installed in the room and a body-mounted wireless accelerometer-based system attached to the person. The proposed system exploits the features of different sensors to maximize the recognition accuracy, improve scalability and thus enhance the reliability. In particular, several instances of a hybrid classifier based on support vector machines and hierarchical temporal memories were used to detect potentially dangerous activities of each person in the environment. If an activity is detected on a specific person wearing the accelerometer, the system localizes and activates it with the aim to receive data and then performs a reliable fall detection using a specifically trained classifier. Apart from surveillance actions for detecting falls, this system may also be used for the assessment of the independence of elderly people or, in rehabilitation, to assist patients during recovery.

A similar research study on a multi-sensor system for detecting falls in home environment using a wearable wireless MEMS accelerometer with on-board fall detection algorithms and a time-of-flight camera has been developed by Diraco *et al.* [63]. A small size and low power consumption board with a microcontroller, an accelerometer and a GSM module, also allowing for use in outdoor scenarios, has been proposed by Fanucci *et al.* [64].

A smartphone-based fall detection system for monitoring the movements of patients able to recognize a fall and to automatically send a request for support has been presented by Abbate *et al.* [65]. The system includes innovative and effective techniques for the recognition of daily life activities that could be erroneously misdetected as falls (e.g., sitting on a sofa or lying on a bed), with the aim of reducing the frequent problem of false alarms.

**Figure 2:**
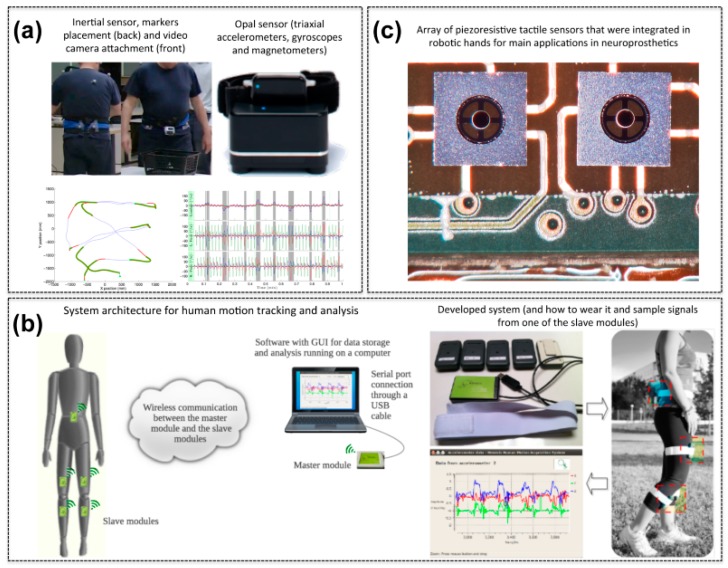
Examples of MEMS for assistance and rehabilitation. (**a**) Wearable inertial sensors for continuous monitoring of turning during spontaneous daily activity. Adapted with permission from [66] (copyright: MDPI—*Sensors* journal); (**b**) Wearable multi-sensor system (composed of a number of small modules that embed high-precision MEMS accelerometers and wireless communications) for human motion monitoring in rehabilitation. Adapted with permission from [72] (copyright: MDPI—*Sensors* journal); (**c**) Silicon MEMS-based piezoresistive sensing array (*i.e.*, four MEMS based piezoresistive sensors) for tactile sensing. (Courtesy of Calogero Maria Oddo).

Monitoring of turning during spontaneous daily activities by using a wearable sensor may represent an important tool for helping clinicians and patients to determine who is at risk of falls with the aim of preventative interventions. In patients with movement disorders, such as PD, turning often results in freezing and/or falling. El-Gohary *et al.* developed an algorithm, using wearable MEMS inertial sensors data, to detect and characterize turns during gait. After a preliminary validation, the turning algorithm was applied to data collected in home from 12 PD and 18 control subjects. The algorithm demonstrated to successfully detects the turn characteristics, and the results showed that, compared to controls, PD subjects are incline to take shorter turns with smaller turn angles and more steps. Furthermore, PD subjects showed more variability in all turn metrics throughout the day and the week [66] (Figure 2a).

A tri-axial accelerometer integrated with humidity, temperature and four pressure sensors was embedded in a less than 4 mm-thick electronic insole for wireless monitoring of motor activities and shoe comfort. Preliminary experiments demonstrated that the device is reliable and may be worn without causing discomfort even for long periods of time, thus suggesting that it may represent a useful device in applications ranging from ergonomics studies on footwear, rehabilitation and also sports [67].

With the specific aim to objectively monitor stroke patient’s upper and lower extremity motor functions in daily life activities and in home training, a complete sensing system has been designed by Klaassen *et al.* within the framework of the European FP7 project INTERACTION [68,69]; it comprises IMU, knitted piezoresistive fabric goniometers, strain sensors, EMG electrodes and force sensors, all integrated into a modular sensor suit.

As previously mentioned, several authors have performed studies to monitor the vital signs of people staying at home with wireless systems employing inertia-based technologies. IMUs have also been integrated in consolidated tools accompanying rehabilitation treatment [70]. An example of a motion capture device based on MEMS inertial sensors, able to provide both an accurate measurement of some motion parameters of a human arm and a graphical reconstruction of the movement on a synthetic model, has been developed by Mirabella *et al.* [71]. The developed caption system can be exploited not only in rehabilitation procedures, but also for sport training. González-Villanueva *et al.* developed a wearable multi-sensor system for human motion monitoring for a specific use in rehabilitation procedures; it is composed of a number of small modules that embed MEMS accelerometers and wireless communication modules to transmit the data related to the body motion to an external acquisition device [72,73] (Figure 2b). The described system has been tested in a Sun Salutation exercise (*i.e.*, a flowing sequence of 12 yoga poses) with a wearable monitoring system composed by five high precision tri-axial MEMS accelerometers and worn by the patient while performing the exercise. Due to the huge amount of available data and complexity of the exercise, a computational system able of interpreting and generating linguistic descriptions about the exercise has been implemented and presented in [74]. Finally, in the framework of the motion tracking system for range of motion measurements in home rehabilitation, an IMU-based system, capable of deriving a real time 3D reconstruction of human posture, has been developed by Daponte *et al.* [75].

MEMS technologies are demonstrating a significant role and applicability in synthetic [76] and bio-artificial [77] tactile sensors towards rehabilitation aims in neuroprosthetics [78], for sensing human-machine interaction [79], and for scientific purposes such as investigating and mimicking the human sense of touch [80,81]. Examples of MEMS based technologies that were investigated in Italy for tactile sensing include capacitive [82], piezoresistive [83] (Figure 2c), piezoelectric [84], optoelectronic [85] transduction principles.

### 2.2. Physical Activities, Safety and Environment Sensing

#### 2.2.1. Sport and Leisure

MEMS-based sensors have recently emerged as a key element in sports science, as well as in several daily life domains related to entertainment and leisure. In this framework, several research efforts have been based on or have strongly benefited from quantitative measurements enabled by different sensing technologies, applied to body segments, working environments or tools. It has been recently argued that MEMS are “conquering” sports, since smart devices are increasingly emerging for monitoring activities in a broad range of sports, as well as for tracking actors’ motions and mapping them onto animation for special effects in movies [86].

Inertial sensors, such as accelerometers and gyroscopes, dominate the stage, with several application domains. Fundamental studies, such as those reported by McCamley *et al.*, aimed at identifying initial contact and final contact instants by means of an IMU positioned over the lower lumbar spine of each participant by a waist belt, and by using *ad hoc* Butterworth filters and algorithms [87]. Similar systems were employed in a number of studies, to directly measure or indirectly estimate sport performance related quantities. A wearable 3D IMU was used to estimate the countermovement jump height, associated to lower limb force, on 28 participants, highlighting the need of compensating accelerations due to trunk rotation, in order to obtain reliable and accurate measures [88]. A similar study was performed by Castagna *et al.*, which assessed vertical jump performance on 20 rugby players by using an accelerometer-based system and by comparing these results with those measured through an optical one [89]. The detection of foot-strike and foot-off instants was also targeted by Bergamini *et al.*, through a trunk-mounted IMU [90]. In this study, the authors aimed at measuring stance and stride duration during the maintenance phase of sprint running. Five amateur and six elite athletes were involved in the study, by acquiring force platform and high speed video camera measurements as reference data. All these studies evidenced the opportunity to efficiently collect information in different sport fields by using inertial sensors, without constraining or limiting athletes’ and coaches’ activities.

**Figure 3:**
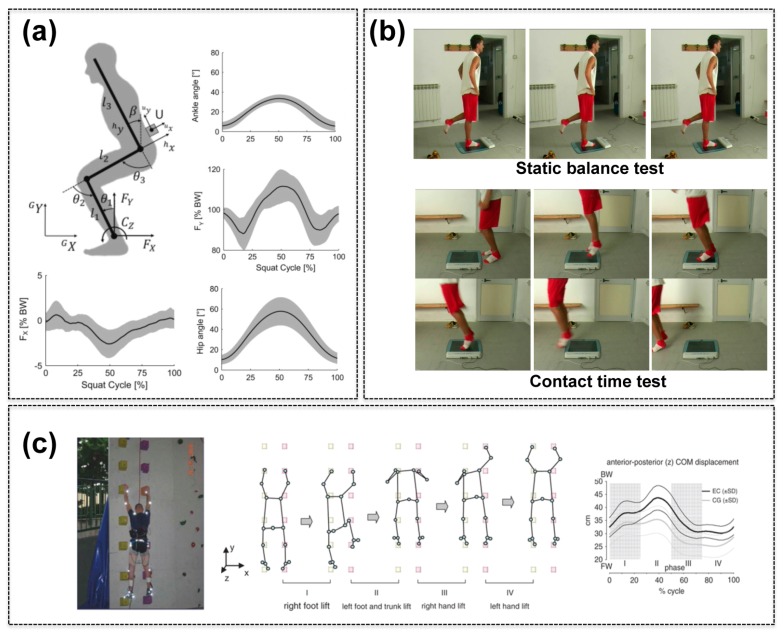
Examples of MEMS for sport and leisure applications. (**a**) IMU mounted on the trunk for estimating squat exercise dynamics. Adapted with permission from [91] (copyright: Elsevier); (**b**) MEMS pressure sensors used to assess balance abilities and non-cyclic rapidity of soccer players. Adapted with permission from [100] (copyright: Creative Commons); (**c**) Climbing dynamics quantified by means of kinematic data associated with vertical plantar reaction forces, measured through MEMS capacitive sensors. Adapted with permission from [104] (copyright: John Wiley & Sons).

Along this line, more specific studies based on inertial sensors have been also reported in the literature. Recently, 10 young healthy volunteers were provided with a single IMU in correspondence to the lower trunk and then asked to perform a squat exercise (10 repetitions). The sensors and a dedicated mechanical model allowed to estimate lower limb joint kinematics, through a least-squares identification algorithm (Figure 3a). The estimates obtained by using inertial sensors were similar to those obtained with a stereophotogrammetric experimental approach in conjunction with the same mechanical model, thus highlighting that the IMU-based approach introduced negligible inaccuracies for this specific application [91]. Masci *et al.* attempted to achieve a more ambitious goal: by using inertial sensors, they tried to quantify developmental differences in the running pattern of children, for both diagnostic and sport training purposes [92]. Running performances were monitored on 54 children (from 2 to 12 years old), revealing that the approach was feasible, although current lacks of fundamental knowledge concerning age related changes in the progression of running performance limited the authors’ conclusion. Inertial sensors placed on the back were also recently proposed to identify skiing techniques, by performing measurements on seven skiers characterized by different skill levels and by using three different sensing systems: Xsens MTw (sampling rate 50 Hz), Humotion (400 Hz) and 2D Datalogger (200 Hz). Interestingly, the analysis of angular velocity cross-plots evidenced differences between differently skilled athletes [93]. However, the limited number of subjects involved in this study highlights the need of further tests to confirm such results.

Networks of inertial sensors, non-conventional IMUs or smartphones and more complex devices, combining inertial sensors with other ones, were tested, allowing in some cases to gain additional insights on different aspects of sport performances. Zanetti *et al.* reported the results obtained by using a SenseWear Armband—provided with accelerometers, temperature, heat flux and galvanic sensors—on 14 rugby players. The aim of this study was to assess the energy expenditure of these athletes during different phases of exercise and recovery. Results revealed that the system was not able to provide a valid estimation of energy expenditure, thus highlighting the need of further improvements of the device [94]. Custom sensor nodes, including a tri-axial accelerometer and a GPS, were developed by Bassetti *et al.* and then tested on Alpine skiers. Qualitative feelings of a professional ski tester were compared with the measured accelerations and trajectories, revealing a good matching between them [95]. 

A Wireless Body Area Network based on a smartphone and integrated with Bluetooth hands free sensors was recently proposed by Depari *et al.* The approach feasibility was demonstrated [96], thus opening the way to less invasive methods respect to chest belts and similar ones, normally used to collect data on subjects’ physical conditions. An interesting approach for quantitatively estimate tremor (associated with fatigue) in sports was recently proposed: the monitoring device was based on a component of the Wii console, the Wiimote, a wireless tri-axial accelerometer which can communicate via Bluetooth with a personal computer. The study revealed that such inertial system was able to observe frequency peaks at 8–10 Hz, which characterize postural tremor, and an increase of tremor intensity after exercise [97]. Moreover, the Wiimote largely outperformed spiralometry and laser pointing systems. Synchronization, which is a key aspect in team sports, was quantitatively estimated by Cesarini *et al.* by means of a network of wireless accelerometers [98], thus highlighting the possibility of successfully monitoring movement synchronism. The same authors recently combined acceleration data (through inertial sensors) and distance travelled (through a GPS) to estimate the kinematic parameters of boat motion during on-water rowing training [99]. Kinematic data were transferred to a smartphone, configured to run a specific application. This made boat acceleration-time-trace audible in real time, thus providing an acoustic feedback to the athletes.

MEMS-based pressure sensors also have a key role in sport science. The main application is related to the monitoring of plantar reaction forces and centre-of-pressure (COP) displacements, for quantifying balance performance. A recent study by Ricotti *et al.* highlighted the correlation between COP recordings and adult soccer players’ skills, by analysing more than 185 subjects playing soccer in teams enrolled in all the ten different Italian soccer leagues [100] (Figure 3b). The same authors had also evidenced the potential of this approach for measuring performance progresses in young athletes [101,102]. COP recordings also allowed to infer differences in basketball player skills: 24 subjects playing basketball at different levels were asked to perform four technical gestures (*i.e.*, free throw, jump stop shot, three-point shot and lay-up) barefoot on a pressure platform, thus allowing to evidence differences in postural parameters, depending on the skill level [103]. The mentioned studies relied on portable—but anyhow bulky—force platforms, which integrate several MEMS pressure sensors.

Recent efforts attempted to develop miniaturized wearable systems to obtain pressure-related outputs. An example of this approach was reported by Zampagni *et al.*, which developed a portable plantar pressure monitoring system, usable during climbing tasks. Nine elite climbers and nine control subjects were analysed. The authors found that expert climbers showed smaller COP oscillations in comparison with the controls (Figure 3c). In addition, a convergence of the optimal solution towards a more diagonal climbing strategy in elite climbers allowed the authors to discuss the origin of the diagonal gait in primates and early hominids habituated to quadrupedal vertical locomotion [104]. Another interesting solution was proposed by Bottoni *et al.*, who used wearable mini paddles provided with MEMS pressure sensors to monitor technical skill differences in swimmers. The authors found that each athlete showed a distinctive shape of the pressure curve, but triathlon swimmers showed a greater variability in the pressure pattern than top level swimmers [105]. Pressure sensors were also recently mounted on the shoulders of front row rugby players, to measure the mechanical loads on professional players of 11 elite rugby teams, when they adopted different strategies during scrummaging. The study evidenced that certain strategies reduced the stresses acting on players, thus representing a possible improvement for players’ safety [106].

Finally, MEMS-based chemical/optical sensors were recently used for monitoring different physiological parameters, in sports. Affinity-based biosensors, for example, are being extensively used in sports medicine and for enhancing selectivity in doping control analyses, as reviewed by Mazzei *et al.* [107]. Wearable systems have been recently reported, with the ability of measuring sweat-related parameters and of integrating these data with additional inputs. Caldara *et al.* recently reported an integrated wearable platform able to simultaneously measure sweat pH by means of a functionalized textile and a colour sensor and skin temperature. Such system may be useful for estimating the body hydration level during exercise or a heat stress [108]. The same authors had also developed a textile-optoelectronic pH meter by combining an organically modified silicate and a novel low power colour sensor, in order to obtain a wearable and reliable sweat analyser. The device exhibited a dynamic pH range from 4.0 to 8.0 and an estimated resolution of 0.05 pH unit [109].

#### 2.2.2. Safety and Environmental Sensing

Smart systems collate a series of leading technologies and solutions aiming at improving safety at work, in buildings, in vehicles, *etc.* and at monitoring environmental conditions. In this framework, MEMS-based sensors play a key role. Often, no design methodologies for smart systems can manage and apply high-level functional constraints at different abstraction levels with a generalized point of view. This approach can be associated with constraint driven design paradigms, predominant in safety and environmental sensing applications [110]. Thus, *ad hoc* devices and sensing strategies must be applied.

Inertial sensors and accelerometer-based devices represent non-invasive and effective solutions able to detect falls in controlled environments; it represents one of the main causes of trauma, disability and death among older people. Studies of researchers, such as Mannini *et al.*, Rescio *et al.* and others [54,111], that might be included in this section have been already described in Section 2.1.2. Assistance and Rehabilitation.

Vehicle safety concerns the possibility to implement automatic urban navigation strategies, which may reduce or eliminate accidents. Angrisano *et al.* recently proposed a low cost combination of GPS and IMUs for vehicular urban navigation. The authors highlighted that inertial sensors are able to provide useful inputs when satellite visibility is poor, with significant advantages in comparison with GPS only integrated systems [112]. Fastellini *et al.* also reported interesting results concerning navigation in difficult urban environments, characterized by many obstructions. Systems based on GPS standalone receivers evidenced a relevant percentage of bad solutions (gaps and outlier) caused by obstructions and multipaths. A low cost MEMS device was able to bridge gaps due to GPS outages, although for limited time spans. These outcomes may allow the design of a vehicle monitoring system installed on a public and scholastic transport fleet [113].

MEMS sensors have been also used to monitor seismic areas and buildings. MEMS acceleration measurements have been compared with linear voltage displacement transducers ones by Trapani *et al*. Results showed that the accuracy of the displacement time histories retrieved by double integration of the acceleration data was of the order of 98%. The authors concluded that, for seismic monitoring, the displacement measurement system based on inertial sensors is better for structures remaining in the elastic range of displacement—so with no residual displacement after the earthquake—while a vision measurement system is more suitable to estimate the residual post-earthquake displacement (if any) of the structure [114]. Twenty eight MEMS accelerometers and evolutionary algorithms were recently used to monitor the dynamic properties of the Manhattan bridge, with the aim of early detecting fatigue phenomena induced by vibrations and distortions [115]. An accelerometer-based system was also used to monitor the structural stability of the tower of the engineering faculty in Bologna, by researchers working in that building [116].

A damage identification method termed “Interpolation Damage Detection Method” was recently proposed and applied to a numerical model of the Shimotsui-Seto bridge by Domaneschi and colleagues. The method allowed to detect localized reductions of stiffness along the bridge deck on the base of accelerometric responses recorded on the main girder during a damaging seismic event, or during an aftershock following the onset of damage. Finite element-based simulations were used to verify the reliability of such monitoring methods, finding promising results [117].

Applications of MEMS in environmental monitoring can be also found in the literature. An interesting example was reported by Andò *et al.*, who microfabricated a triple-bent-beam contactless temperature sensor. Electroplated nickel cascaded bent beams were developed by using a high-aspect-ratio process called MetalMUMPs, over a silicon nitride isolation layer. Sensor behaviour was modelled both analytically and numerically, and experimental characterization was then performed. An integrated inductor was also designed to achieve a LC resonator coupled with a remote readout circuit to sense the resonance frequency shift in response to temperature variation [118].

Gas sensors are also of primary important for environmental monitoring. Portable methane sensors were recently developed by coupling a MEMS cantilever with a low temperature co-fired ceramic differential photo acoustic cell and a spatial interferometer. Four different gas chambers were included in the system; namely, a sample cell, a reference cell and a differential photo acoustic cell composed of two parts: a sample beam chamber (SBC) on top and a reference beam chamber (RBC) at the bottom. The MEMS cantilever microphone was located between the SBC and RBC chambers, both filled with the target gas. The sensor showed high sensitivity to radiation whose optical wavelengths corresponded to the absorption lines of the target gas [119].

A high sensitivity humidity sensor was recently proposed by Orsini *et al.* The device was obtained through a hydrothermal process: aluminium thin film micro-patterned tracks were deposited on a glass substrate and modified via wet chemistry processes, in order to transform the aluminium metal into Zn/Al layered double structures. This approach showed good performance and high sensitivity at room temperature, and was CMOS-compatible and low power [120].

When monitoring must be performed in harsh environments, not only sensors, but also conditioning and memorization systems must be properly designed. Also in this framework, MEMS open important possibilities. For example, MEMS-based non-volatile memory arrays were recently developed by assembling a NanoMech^TM^ MEMS switch from Cavendish Kinetics (‘s-Hertogenbosch, The Netherlands), designed by using a 0.35 μm CMOS technology with four metal layers, and proposed for applications in extreme environments [121].

## 3. Conclusions and Future Perspectives

This review paper, inspired by the Micromachine Summit events organized by the Micromachine Center (MMC) of Japan in which the authors usually participate, reports significant examples of research oriented applications in Italy during the last 3 years, in which MEMS sensor technologies played a central role. Key application drivers, such as healthcare, wellness and environment monitoring pushed forward the development of innovative solutions with the purpose to improve the quality of life in a pervasive network environment.

Novel approaches and strategies based on MEMS and BioMEMS technologies have increased significantly over the past 30 years the availability of applications and, thanks to the establishment of wireless communications, set the stage for the development of wireless sensor networks for healthcare, well-being, assistance, rehabilitation and ambient/environmental sensing.

The emergence of MEMS sensors able to efficiently and reliably monitor physical parameters can change the paradigm of healthcare and well-being; MEMS sensors can support personalized medicine and can be used to identify early warning signs and to predict and manage specific events, thus intervening before problems arise [1,2].

Nowadays, as presented in this paper that reports only the Italian “local” scenario, MEMS sensors are being integrated into a huge number of solutions for healthcare, well-being and environmental monitoring applications. Together with the establishment and advancements in mobile and cloud computing and pervasive communications (which are actually significant fields of research), MEMS sensor technologies can take part of a paradigm shift, from a “current reactive healthcare model” to a “wellness preservation model”. The role of MEMS sensor technologies and derivative solutions will continue to grow up in the coming decades, thanks to the falling cost of sensor technologies and advancements in infrastructural and information and communications technologies, leading to an efficient and effective aggregation, processing, storage and visualization of sensor data [122].

**Figure 4:**
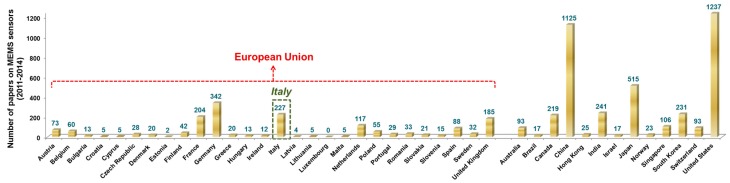
Number of research papers published in the period 2011–2014 on MEMS sensors. The analysis was conducted for all the EU member states and for other countries with relatively high income and technological development level. Source: Scopus, searching the word “MEMS sensor” in title, abstract and keywords for journal papers and conference proceedings.

It is interesting to compare the recent research outputs that Italy produced on these topics with those emerged in other countries, worldwide. Figure 4 shows the number of research papers published on MEMS sensors, in the period 2011–2014, for all the countries that are members of the European Union (EU) and for other high-income and emerging countries.

Within the EU, Italy scores second in terms of papers published on MEMS sensors (227), after Germany (342). France, United Kingdom and The Netherlands follow Italy with a comparable but smaller scientific production. On a global level, United States and China dominate the stage, with 1237 and 1125 published papers, respectively, followed by Japan, with 515. The scientific production of other world-leading and emerging countries on this topic, such as South Korea, Canada and India, is comparable to Italy’s, thus confirming the leading role of Italy, at a global level, in the multifaceted research fields related to MEMS sensors.

Future trends and research/industrial perspectives may consider further integration of MEMS sensor technologies within devices and objects already used on a daily basis by people (e.g., smartphones); it would represent a kind of “everyday technology” for routinely monitoring activity and health, more and more in a multisensing and multimodal cloud framework [123]. The integration of MEMS sensor-based technologies would enable the continuous and integrated management of a huge number of different information, representing a smart ecosystem that is intended to “cohabit” and improve together with the enhancement of the computational performances of microprocessors. In this framework, as mentioned by the company STMicroelectronics, one of the main future drivers is represented by the possibility to maintain the sensors always on, in order to continuously monitor the users’ activity (also indoor without the need of a GPS) and environment conditions. Energy consumption, one of the main consequence of the aforementioned scenario, is an open research issue that has to be approached, as again mentioned by STMicroelectronics, with sensors even more integrated and that will implement, in a single device, smart power management and signal processing systems [124].

Sensor technologies have to be more transparent to users and be part of a comfortable and non-impact environment, such as smartphones, tablets and clothes for offering better performance, less invasiveness, more predictability and extensibility. Reducing the requirement for conscious human action in the sensing process (e.g., calibration of devices) by means of well consistent and reliable detection, classification and prediction techniques will also represent a needed improvement to be addressed. In this framework, progress in the direction of high performance computing, cloud computing, big data analyses and so on will provide the key tools to make those scientific and technological breakthroughs possible [123].

The rate of advancement in MEMS technologies is expected to accelerate [125], driven also by the growing commercial demand to understand and manage personal health and well-being and monitoring of environmental conditions [126]. New MEMS sensors will have higher selectivity and sensitivity also with a higher stability and capability to classify the events and avoid false alarms [123]. 

An important trend of future MEMS sensor technologies with significant and promising scientific and industrial perspectives is the advancement of non-contact physiological sensing (e.g., through the increased materials flexibility), for avoiding a direct contact with the human body and thus reducing specific usability constraints.

It is worth mentioning that while MEMS sensor technologies for measuring physical variables are standardized, they lag behind in chemical and gas sensing applications. In this framework, by exploiting new molecular-based sensing elements and biosensor technologies, *i.e.*, BioMEMS, the capability to early identify biochemical disease markers and chemical/biological contaminants will be improved. Therefore, potential research and development of new sensing principles may represent future scientific and technological breakthroughs with an immediate effect also on industries; the continuous advancements in MEMS technologies and evolution in semiconductor manufacturing techniques will enable the development and production of MEMS sensors in large quantities, cost effectively and with a high reliability and stability, based on electrochemical, chemo-optical, and kinematic sensing principles.

In the “local” scenario of Italy, the country where the handicraft originated, MEMS sensors and modular development solutions and technologies, such as the development boards produced by STMicroelectronics (French/Italian multinational electronics and semiconductor manufacturer headquartered in Geneva, Switzerland [40]) and the Arduino technologies (originated in Ivrea, Italy [127]) may represent new tools for the new “Italian handicraftsman” to enable and invent novel smart sensor-based technologies. In this framework, such approaches/trends will represent a paradigm change in the drive for further improvements in technologies: “innovation based on the needs of the community with respect to standardized offerings, towards high impact local applications and resources for a global technology improvement” [128].
